# Morphological, electrophysiological, and molecular alterations in foetal noncompacted cardiomyopathy induced by disruption of ROCK signalling

**DOI:** 10.3389/fcell.2024.1471751

**Published:** 2024-10-07

**Authors:** David Sedmera, Veronika Olejnickova, Barbora Sankova, Hana Kolesova, Martin Bartos, Alena Kvasilova, Lauren C. Phillips, Simon D. Bamforth, Helen M. Phillips

**Affiliations:** ^1^ Institute of Anatomy, First Faculty of Medicine, Charles University, Prague, Czechia; ^2^ Laboratory of Developmental Cardiology, Institute of Physiology, Czech Academy of Sciences, Prague, Czechia; ^3^ Institute of Dental Medicine, First Faculty of Medicine, Charles University, Prague, Czechia; ^4^ Biosciences Institute, Newcastle University, Newcastle upon Tyne, United Kingdom

**Keywords:** mouse embryonic heart, ventricular wall, myocardial trabeculae, compaction, conduction, cardiomyocyte proliferation, ROCK

## Abstract

Left ventricular noncompaction cardiomyopathy is associated with heart failure, arrhythmia, and sudden cardiac death. The developmental mechanism underpinning noncompaction in the adult heart is still not fully understood, with lack of trabeculae compaction, hypertrabeculation, and loss of proliferation cited as possible causes. To study this, we utilised a mouse model of aberrant Rho kinase (ROCK) signalling in cardiomyocytes, which led to a noncompaction phenotype during embryogenesis, and monitored how this progressed after birth and into adulthood. The cause of the early noncompaction at E15.5 was attributed to a decrease in proliferation in the developing ventricular wall. By E18.5, the phenotype became patchy, with regions of noncompaction interspersed with thick compacted areas of ventricular wall. To study how this altered myoarchitecture of the heart influenced impulse propagation in the developing and adult heart, we used histology with immunohistochemistry for gap junction protein expression, optical mapping, and electrocardiography. At the prenatal stages, a clear reduction in left ventricular wall thickness, accompanied by abnormal conduction of the ectopically paced beat in that area, was observed in mutant hearts. This correlated with increased expression of connexin-40 and connexin-43 in noncompacted trabeculae. In postnatal stages, left ventricular noncompaction was resolved, but the right ventricular wall remained structurally abnormal through to adulthood with cardiomyocyte hypertrophy and retention of myocardial crypts. Thus, this is a novel model of self-correcting embryonic hypertrabeculation cardiomyopathy, but it highlights that remodelling potential differs between the left and right ventricles. We conclude that disruption of ROCK signalling induces both morphological and electrophysiological changes that evolve over time, highlighting the link between myocyte proliferation and noncompaction phenotypes and electrophysiological differentiation.

## 1 Introduction

Myocardial noncompaction has been recognised as a distinct form of cardiomyopathy (Dusek et al., 1975), but recent studies (Faber et al., 2021a) have questioned the developmental mechanism of the disease. While it is traditionally described as a deficit of trabecular compaction, it could also be regarded as a problem of compact myocardium thickening (Jensen et al., 2024) based on deficient myocyte proliferation, with hypertrabeculation manifesting as a secondary, compensatory feature. Myocardial noncompaction has been described in many different gene defects [reviewed, e.g., by Sedmera et al. (2000) and Wessels and Sedmera (2003)], and also after drug treatment (Krejci et al., 2016; Cao et al., 2019). Analysis of clinical pedigrees shows heterogeneity of phenotypes among individuals sharing the same mutation, and incomplete penetrance is a rule rather than an exception (Jenni et al., 2001; Varnava, 2001; Paterick and Tajik, 2012). This, therefore, suggests that the underlying developmental mechanism of noncompaction is complex and may vary between individuals.

The speed of electrical impulse propagation depends on myocardial architecture (i.e., myocyte length-to-width ratio, fibrosis, and myocardial gap junctional coupling), and disruption of this structure by various disease conditions, including noncompaction, can impact ventricular conduction. The role of the myocyte organisation itself on impulse spreading was demonstrated on variously patterned myocyte cultures *in vitro* (Kucera et al., 1998). The importance of interaction with the fibroblasts was shown in the co-cultures (Rohr, 2012), and fibrosis is a known factor in the formation of arrhythmogenic substrates (Akar et al., 2004; Handa et al., 2021). Changes in the expression and organisation of gap junctional proteins were also reported in various models (Kostin et al., 2003; Boulaksil et al., 2010; Sedmera et al., 2016; Andelova et al., 2021). However, the links between compact myocardium structure, myocardial architecture, gap junctional protein expression, and cardiac conduction have not been clearly elucidated yet.

To address this, we utilised Rho-kinase (*ROCK*) transgenic mouse lines as a model to study the relationship between the myocardial architecture of the developing cardiac ventricular wall and impulse propagation through the foetal and adult heart. ROCK is a serine/threonine protein kinase with two isoforms, ROCK1 and ROCK2, which function as downstream effectors of the small GTPase RhoA. Activation of RhoA leads to the binding of GTP-RhoA to the Rho-binding domain of ROCK, triggering numerous downstream pathways that affect diverse cellular functions such as contraction, cell migration, and actin cytoskeleton organisation. A role for ROCK signalling in cell cycle progression has been implicated in tumorigenesis (Kumper et al., 2016) and the heart (Zhao and Rivkees, 2003).

The *ROCK* genes are expressed from the beginning of heart development in the cardiac crescent, are required for the migration of cardiac precursor cells at embryonic day (E)7.5 (Wei et al., 2001; Zhao and Rivkees, 2003), and continue to be expressed as the heart develops (Thumkeo et al., 2003; Shimizu et al., 2005). The global deletion of *ROCK1* or *ROCK2* (Thumkeo et al., 2003; Shimizu et al., 2005; Zhang et al., 2006), and the cardiomyocyte-specific simultaneous deletion of both *ROCK1* and *ROCK2* using *MHC-Cre* or *Nkx2.5-Cre* (Shi and Wei, 2022) are all embryonic lethal. Numerous studies have utilised different adult transgenic ROCK mouse models and have demonstrated different roles for each isoform with pathological-induced conditions. ROCK2 plays a role in regulating cardiac hypertrophic responses (Okamoto et al., 2013; Ikeda et al., 2014; Sunamura et al., 2018), whereas ROCK1 is involved in the pathogenesis of fibrosis (Zhang et al., 2006) and the transition from cardiac hypertrophy to heart failure (Shi et al., 2010).

We used the well-established *ROCK* dominant-negative (*ROCKDN*) transgenic mouse model, which we and others have successfully used to specifically downregulate the cellular function of both ROCK isoforms using *Cre*-mediated recombination (Amano et al., 1998; Kobayashi et al., 2004; Kobayashi et al., 2011; Phillips et al., 2012; Phillips et al., 2013; Ikeda et al., 2014; Ellawindy et al., 2015). Under the control of Cre recombinase, the ROCKDN construct expresses a dominant-negative ROCK protein that binds to and disrupts the function of both endogenous ROCK proteins as it suppresses the Rho-binding activity (Amano et al., 1998; Amano et al., 1999). We generated *ROCKDN* ^*Gata5-Cre*^ transgenic mice to disrupt ROCK signalling from E9.25 in the developing ventricular wall and monitored the structural impact on the maturation of the ventricular wall and how this affected the electrophysiology of the heart through optical mapping. We also crossed these mice with the *Cx40:eGFP* mouse line to label the cardiac ventricular conduction system (Miquerol et al., 2004) and used optical mapping *ex vivo* to study ventricular activation in spontaneous and paced rhythms. Here, we report the structure–function relationship between abnormal foetal hypertrabeculation, connexin (Cx)40 and −43 expression, and ventricular activation patterns in the prenatal as well as postnatal stages and highlight the ability of the heart to recover from early structural abnormalities.

## 2 Materials and methods

### 2.1 Animals

All mice used in this study have been described elsewhere: *ROCK* dominant-negative (*ROCKDN*) mice (Kobayashi et al., 2004) were intercrossed with *TnT-Cre* to target cardiomyocytes from E7.5 (Jiao et al., 2003) or *Gata5-Cre* (Merki et al., 2005) to target downregulation of ROCK function within the myocardium and epicardium from E9.25. To visualise Gata5-Cre expression patterns within the heart, *Gata5-Cre* males were crossed with the reporter line *R262R* ^*eyfp*^ (Srinivas et al., 2001). To study the conduction system, the *ROCKDN* ^*Gata5-Cre*^ line was crossed with the *Cx40:eGFP* knock-in model (Miquerol et al., 2004) to visualise the expression of Cx40 in the ventricular myocardium. Due to the nature of this transgene, which effectively disrupts the endogenous Cx40 protein, only *Cx40:eGFP* heterozygotes were used, and they were used solely for morphological evaluation, as some minimal electrophysiological phenotype is evident in the prenatal stages (Sankova et al., 2012; Benes et al., 2014).

*ROCKDN* heterozygous females were crossed with a *TnT-Cre* or *Gata5-Cre* male to generate heterozygous mutant mice (*ROCKDN* ^*+/−;Cre*+ve^, referred to as *ROCKDN* ^*TnT-Cre*^ or *ROCKDN* ^*Gata5-Cre*^) and littermate controls (*ROCKDN* ^*+/−;Cre*-ve^, *ROCKDN* ^*−/−;Cre*+ve^ *ROCKDN* ^*−/−;Cre*-ve^). Male and female mice were mated, and the identification of a vaginal plug was deemed as E0.5. For the collection of embryos, the pregnant females were culled on the required day of embryonic development.

Mice were genotyped by standard PCR using previously published allele-specific primers for each mouse line. All studies involving animals were performed in accordance with the UK Home Office Animals (Scientific Procedures) Act 1986.

### 2.2 Cardiac imaging

E15.5 embryos were collected (*n* = 5), processed, and analysed by high-resolution episcopic microscopy (HREM) imaging as previously described (Weninger and Mohun, 2007).

Hearts from 15-month-old mice (*n* = 4 per group) were fixed after optical mapping, contrasted with phosphotungstenic acid, and imaged on a Bruker micro-CT scanner. Image processing and volume rendering were performed as described (Bohuslavova et al., 2019).

### 2.3 RNAscope

*ROCK1* and *ROCK2* mRNA expression was examined by RNA *in situ* hybridisation using an RNAscope Multiplex Fluorescent v2 Assay (Advanced Cell Diagnostics, Newark, CA, United States) following the manufacturer’s instructions. The targeting probes Mm-Rock1-C2 (catalogue number 526271-C2) and Mm-Rock2 (catalogue number 507971) were used. Following the RNAscope assay, sections were stained by immunofluorescence.

### 2.4 Histology and immunofluorescence

Embryos and hearts collected for analysis and those used for optical mapping were fixed by immersion in 4% paraformaldehyde in phosphate buffer saline (PBS, pH = 7.4) for 24 h at 4°C. For cardiomyocyte proliferation analysis, pregnant dams received a single intraperitoneal injection of BrdU (100 mg/kg at 10 mg/mL) 1 h prior to sacrifice. After processing to paraffin, serial sections were cut at 10 µm and alternatively stained with H&E/Picrosirius Red or by immunofluorescence following standard histology protocols. Sections were incubated with the following selected primary antibodies: rat anti-BrdU (Abcam, Cambridge, United Kingdom; ab6326), rabbit anti-GFP (Torrey Pines; TP401), mouse anti-MF20 (Developmental Studies Hybridoma bank; MF20-c), rat anti-CD31 (BD Biosciences, San Jose, CA, United States; 553370), rabbit anti-Cx43 (Sigma; C6219), and rabbit anti-GFP (Abcam, Cambridge, United Kingdom; ab6556). For fluorescence visualisation, cell boundaries were counterstained with wheat germ agglutinin (WGA) coupled with Alexa594 and the nuclei were stained with DAPI or Hoechst 33342, as described (Benes et al., 2011). Images were acquired on an AxioImager (Zeiss), confocal images were acquired on a Nikon AIR confocal or on an Olympus FluoView upright confocal system, and transmitted light images were obtained on an Olympus slide scanner. To assess proliferation, the number of BrdU-positive cells was divided by the total number of cells in each region of interest (*n* = 3 embryos per genotype; *n* = 3 sections per heart). Cell counting was performed using ImageJ software (National Institutes of Health, Bethesda, MD, United States) using the cell counter feature. To assess Cx43 staining, maximum intensity projection images were taken from four confocal sections, each 1 µm apart. Using ImageJ software, the intensity of the staining was converted to a percentage by comparing against the myocardial tissue autofluorescence as a proxy of total tissue volume.

### 2.5 Ventricular wall thickness measurements

The compact wall from transverse sections of hearts in a four-chambered view, from histology and HREM images, was measured using ImageJ (*n* = 3 sections per heart). The investigator was blinded to the sample genotype.

### 2.6 qPCR

Total RNA was isolated from embryonic hearts using the ReliaPrep™ RNA Miniprep kit (Promega). cDNA was synthesised using the high-capacity cDNA reverse transcription kit (ThermoFisher Scientific). Primers were designed to amplify *p21* (5′-TGA​ATG​GAG​ACA​GAG​ACC​CC and 5′-ATA​GAA​ATC​TGT​CAG​GCT​GGT​C) and the reference gene *Gapdh* (5′-TGT​GCA​GTG​CCA​GCC​TCG​TC and 5′-TGA​CCA​GGC​GCC​CAA​TAC​GG). qPCR was performed using SYBR green master mix (ThermoFisher Scientific, in triplicate on a QuantStudio 7 Real-Time PCR System). Data were analysed using the comparative Ct method (Schmittgen and Livak, 2008) and were normalised to *Gapdh*.

### 2.7 Optical mapping

Pregnant females were sacrificed at E15.5 and E18.5, and the embryos were placed in ice-cold oxygenated Tyrode’s buffer. Following the removal of the embryonic membranes, the crown-rump length was measured, and the embryos were killed by rapid decapitation. The hearts with adjacent posterior body walls were isolated and stained by immersion in di-4-ANEPPS solution for 12 min on ice as described (Sankova et al., 2012). Heart activation patterns were evaluated using the ×4 objective, and spontaneous vs. stimulated left ventricular activation was quantified using a water-dipping ×10 lens. Analysis of the recordings was performed as described (Olejnickova et al., 2018).

Optical mapping of isolated adult hearts (12 months and 15 months) was performed using a standard perfusion system as described (de la Rosa et al., 2013; Sedmera et al., 2016).

### 2.8 Electrocardiography (ECG)

ECG traces were measured in adult mice using a three-lead ECG as described (Sysa-Shah et al., 2015), and analysis was performed using LabChart software (AD Instruments).

### 2.9 Statistical analysis

A normality test (Shapiro–Wilk) was used to assess the distribution of data. Then, the appropriate test, either an unpaired t-test or a one-way ANOVA with Bonferroni correction for multiple comparisons, was performed to determine statistical significance. A chi-square test was used to test for Mendelian inheritance. All data are presented as mean ± SEM. *p* < 0.05 was considered statistically significant. Analyses were performed using GraphPad Prism software (version 9.3.1).

## 3 Results

### 3.1 Loss of ROCK function in cardiomyocytes causes an embryonic noncompaction phenotype due to reduced proliferation

**FIGURE 1:**
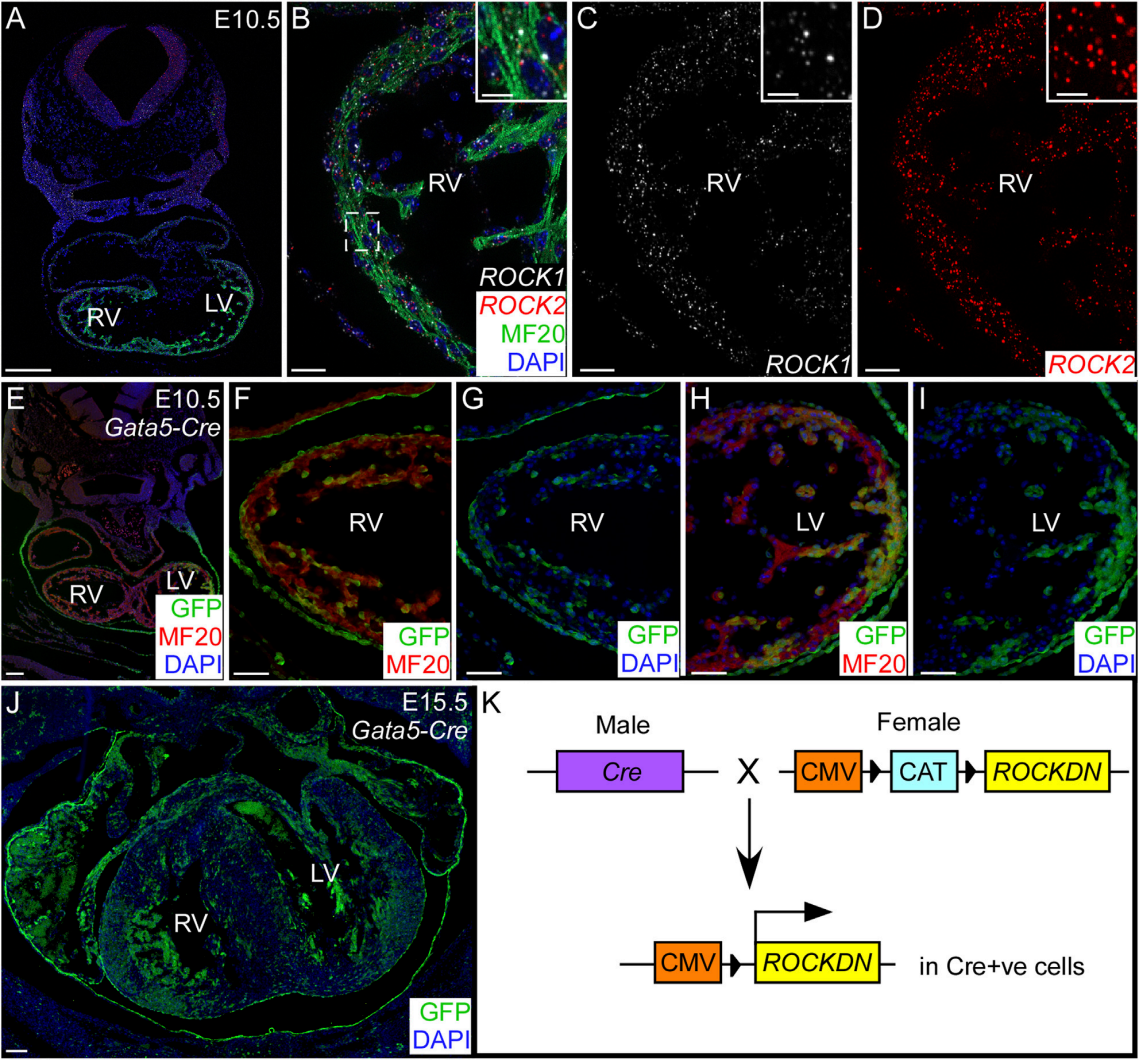
Cardiomyocyte expression of *ROCK1* and *ROCK2* genes and Gata5-Cre within the developing ventricles. **(A–D)** Using RNAscope *in situ* hybridisation on control embryos at E10.5, *ROCK1* (white) and *ROCK2* (red) genes were both expressed in the cardiomyocytes (MF20 antibody, green) throughout the developing ventricles. Inset **(B–D)** shows a high power image of the compact ventricular wall indicated by the dotted box **(C)**. **(E–J)** *Gata5-Cre* mice were crossed with the *R26R* ^*eyfp*^ reporter mouse, and Cre-positive cells were identified by GFP antibody (green). There was patchy staining throughout the cardiomyocytes (MF20, red) in the ventricles, shown at E10.5 **(E–I)** and E15.5 **(J)**. The blue channel shows the nuclear staining (DAPI). **(K)** *Cre* male mice were crossed with *ROCKDN* females in which the expression of ROCKDN was inhibited by the presence of a CAT box. Under the control of the cell-specific expression of Cre, the CAT box was removed, allowing expression of the ROCKDN construct, hence downregulating ROCK function in Cre-activated cells. RV = right ventricle, LV = left ventricle. Scale bar = 200 µm **(A)**, 20 µm **(B–D)**, 2 µm (inset), 100 µm **(E, J)**, 50 µm **(F–I)**.

The initial aim of this study was to establish the impact of deleting the ROCK genes in cardiomyocytes on ventricular wall development. First, we showed, by RNAscope, that during normal cardiovascular development, *ROCK1* and *ROCK2* were both expressed in the cardiomyocytes throughout the ventricular wall of the embryonic heart (Figures 1A–D). To disrupt the function of both *ROCK* genes, we utilised the *ROCKDN* transgenic mouse, which allows the Cre-mediated overexpression of the dominant-negative ROCK (ROCKDN) protein (Kobayashi et al., 2004). The ROCKDN protein dimerises with the endogenous ROCK1 and ROCK2 proteins to inhibit the function of the kinase domains; hence, they are no longer able to bind RhoA. Thus, the ROCK function is blocked in all cells expressing Cre (Figure 1K). To target cardiomyocytes, the *ROCKDN* mouse was crossed with the *TnT-Cre* mouse (Jiao et al., 2003). *Tnt-Cre* is expressed in cardiomyocytes from E7.5 (Supplementary Figures 1A–I). *ROCKDN* ^*TnT-Cre*^ mutants died from E16.5, and no mutants were observed after birth. The ventricular wall was thin and lacked a compact layer, and there was retention of the trabeculae (Supplementary Figures 1J–R).

To allow further investigation of the function of ROCK in cardiomyocytes in the ventricular wall, through development and after birth, the *ROCKDN* mice were crossed with the *Gata5-Cre* mouse (Merki et al., 2005) (generating *ROCKDN* ^*Gata5-Cre*^ mutant mice). *Gata5-Cre* is expressed from E9.25, is maintained throughout development, and is known to have patchy uneven expression throughout the myocardium. Thus, we hypothesised this would produce a less severe phenotype. To confirm this expression, the *Gata5-Cre* mouse was crossed with the *R26R* ^*eyfp*^ reporter mouse, hearts from E10.5 and E15.5 embryos were sectioned, and the Cre-positive cells were stained using a GFP antibody (Figures 1E–J). The specific pattern of expression varied between embryos, but the average percentage of ventricular wall expressing GFP was consistent (E10.5: 60% right ventricle (RV), 45% left ventricle (LV); E15.5: 61% RV, 52% LV, *n* = 3). Thus, in *ROCKDN* ^*Gata5-Cre*^ mutant mice, the ROCK function was disrupted in approximately half of the cardiomyocytes and the epicardium from E9.25.

**FIGURE 2:**
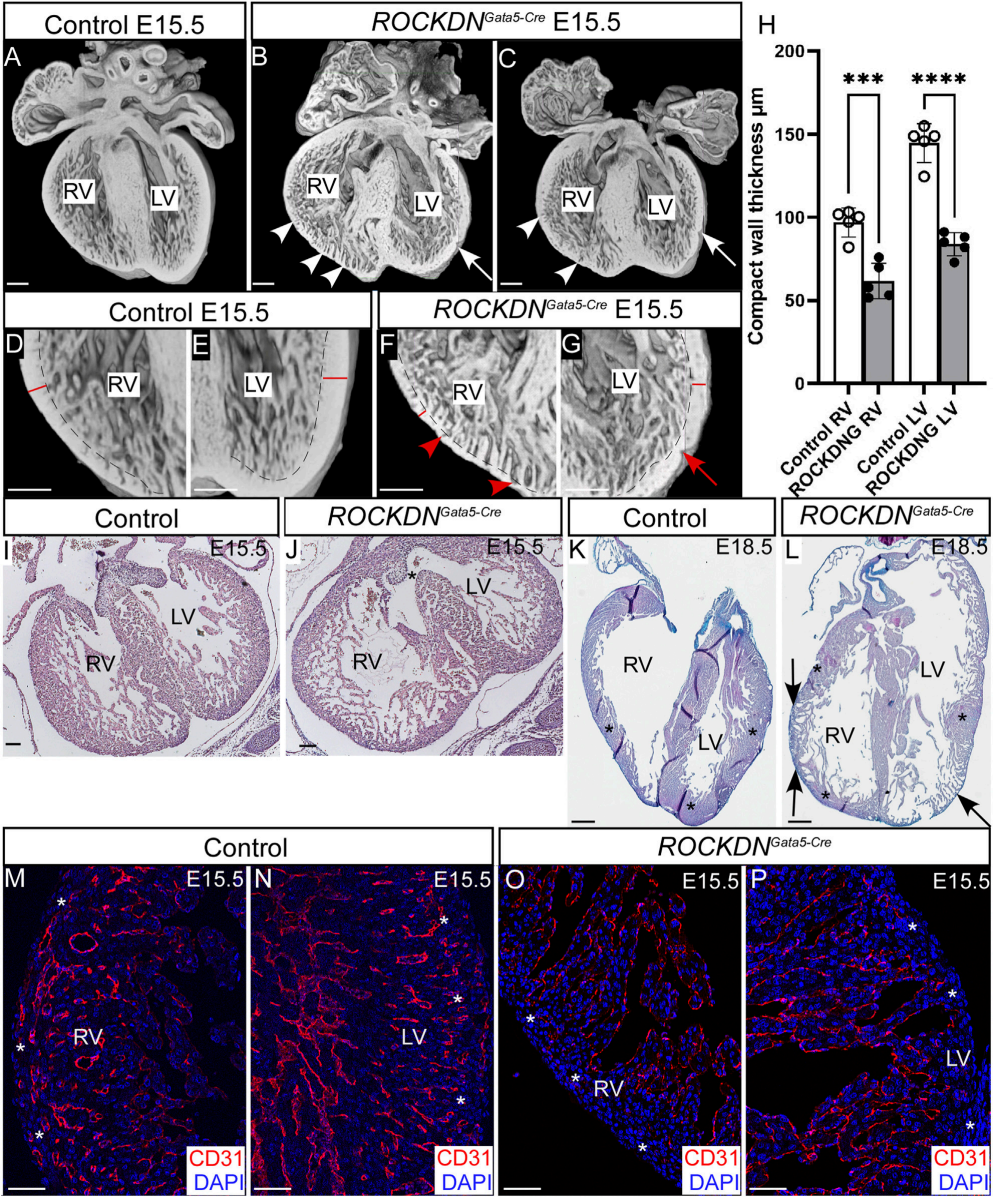
Downregulation of ROCK1 and ROCK2 led to developmental ventricular wall defects in *ROCKDN* ^*Gata5-Cre*^ mutants. **(A–G)** 3D reconstructions were made from E15.5 hearts from control and *ROCKDN* ^*Gata5-Cre*^ mutants using HREM. The compact ventricular walls of the control hearts **(A, D, E)** were uniform, and there was a compact wall (red line) and an inner layer of trabeculae (separated by a dotted line). In the *ROCKDN* ^*Gata5-Cre*^ mutants **(B, C, F, G),** the compact walls were thinner and pinched in places (arrows) with deep trabecular myocardial crypts (arrowheads). **(H)** At E15.5, the compact myocardium of the right and left ventricles was significantly thinner in the *ROCKDN* ^*Gata5-Cre*^ mutants than the control hearts (*n* = 5). **(I, J)** H&E staining at E15.5 showed the presence of a VSD [asterisk in **(J)**] in 16.6% of the *ROCKDN* ^*Gata5-Cre*^ mutants. **(K, L)** At E18.5, a partial noncompaction phenotype of the persistence of the thin compact wall was seen in 33.3% of the *ROCKDN* ^*Gata5-Cre*^ mutants [arrows in **(L)**], adjacent to recovered regions of the normally compacted thickened myocardium [asterisks in **(K, L)**]. **(M–P)** Immunofluorescent staining with CD31 (red) showed normal maturation of coronary arteries at E15.5 within the compact myocardium [white asterisks **(M, N)**], which was missing in the ventricular wall of the *ROCKDN* ^*Gata5-Cre*^ mutants [white asterisks **(O, P)**] (*n* = 3). The blue channel shows the nuclear staining (DAPI). Data are represented as mean ± SEM. ***p < 0.001, ****p < 0.0001 by one-way ANOVA with Bonferroni correction for multiple comparisons. RV = right ventricle, LV = left ventricle, ROCKDNG = *ROCKDN* ^*Gata5-Cre*^ mutants. Scale bar = 200 µm **(A–G)**, 100 µm **(I, J)**, 1 mm **(K, L)**, 50 µm **(M–P)**.

Embryos were collected at E15.5, and the hearts were dissected from control and littermate *ROCKDN* ^*Gata5-Cre*^ mutants. To visualise the overall external and internal structure of the hearts, 3D reconstructions were made using HREM. This showed that the ventricular walls of control embryos were uniform, with a thick compact wall (Figures 2A, D, E). In comparison, the ventricular walls of the mutants were visibly abnormal and had a noncompaction phenotype (Figures 2B, C, F, G). The compact wall was uneven and “pinched” in places (arrows in Figures 2B, C, G), and there were prominent trabeculae with unusual deep crevices, known as myocardial crypts, present in the right ventricular wall and to a lesser extent in the left ventricle (arrowheads in Figures 2B, C, F). Due to the patchy expression pattern of *Gata5-Cre*, the severity of the thin ventricular wall phenotype and positioning of the noncompacted trabeculae varied slightly between mutants (Figures 2B, C). Measurements of the compact myocardium confirmed that the right and left ventricular compact walls were significantly thinner in the *ROCKDN* ^*Gata5-Cre*^ mutants than the control E15.5 hearts (Figure 2H, *n* = 5). Further investigation of the phenotype at E15.5 by histology (*n* = 24) revealed that 83.3% (*n* = 20) of the *ROCKDN* ^*Gata5-Cre*^ mutants had a thin myocardium and persisting trabeculae throughout the developing walls, 66.7% (*n* = 16) had a “pinched” compact myocardium (Figure 2G, red arrow) and 16.6% (*n* = 4) had a ventricular septal defect (VSD) (Figures 2I, J, asterisk). By E18.5, the phenotype was more variable between *ROCKDN* ^*Gata5-Cre*^ mutants, as approximately two-thirds of the foetuses now showed a left ventricular structure indistinguishable from controls. However, one-third of *ROCKDN* ^*Gata5-Cre*^ mutants displayed a localised partial noncompaction phenotype in both ventricular walls of retained trabeculae with a thin compact wall (arrows in Figure 2L), adjacent to regions of normally compacted thick ventricular wall (asterisks in Figures 2K, L).

To further examine the impact of the noncompaction phenotype at E15.5, the overall maturation of the ventricular wall was investigated by CD31 antibody staining, which is expressed in the endothelial cells of both veins and arteries and in endocardial cells. At E15.5, the coronary arteries have formed throughout the outer compact myocardium in the right and left ventricles in the control embryos (Figures 2M, N), which are derived from the first coronary vascular population (Tian et al., 2014). However, in the *ROCKDN* ^*Gata5-Cre*^ mutants, CD31 staining was only visible in the endocardial cells and was absent in the thinner compact myocardium layer (white asterisks in Figures 2M–P), hence suggesting there was a delay in the maturation of the ventricular wall as it remained thin.

**FIGURE 3:**
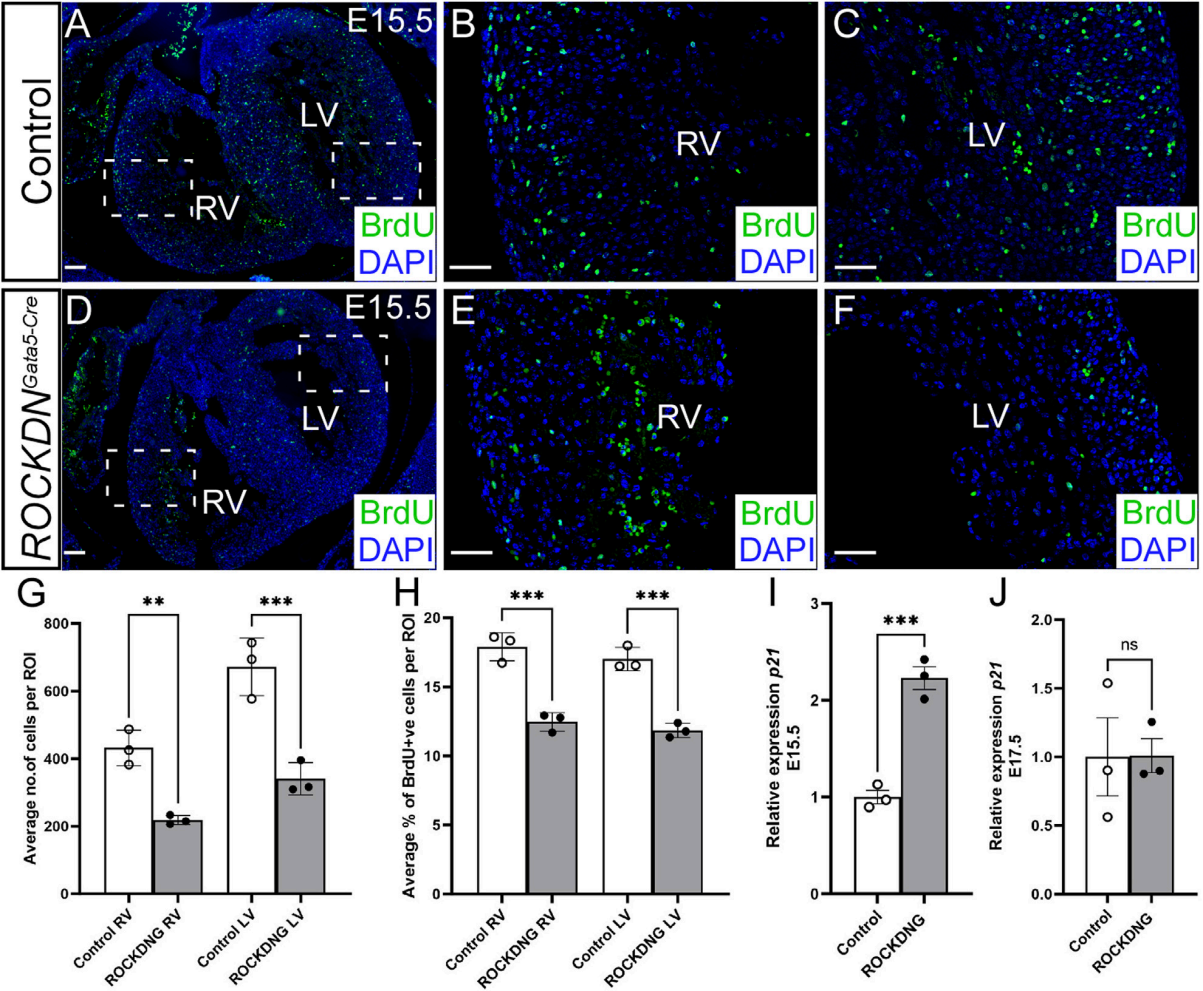
Deficient proliferation correlates with a thin ventricular wall at E15.5 in *ROCKDN* ^*Gata5-Cre*^ mutants. **(A–F)** Pregnant females were injected with BrdU prior to collecting the embryos. Transverse sections from E15.5 control and *ROCKDN* ^*Gata5-Cre*^ mutants (*n* = 3) were stained with BrdU antibody to label the proliferating cells (green) and counterstained with DAPI (blue) to give the total cell count per image. The average total number of cells (all DAPI + ve nuclei) per region of interest (ROI) was significantly reduced in the *ROCKDN* ^*Gata5-Cre*^ mutants **(G)**. This correlated with a corresponding significant reduction in the percentage of proliferation (% of DAPI + ve nuclei, which were BrdU + ve) in both the right and left ventricles **(H)**. **(I, J)** Expression of *p21* in embryonic hearts was measured by qPCR, and there was a significant increase in expression at E15.5 **(I)** but not at E17.5 **(J)** (*n* = 3). Data are represented as mean ± SEM. **p < 0.01, ***p < 0.001, by one-way ANOVA with Bonferroni correction for multiple comparisons **(G, H)** or unpaired t-test **(I, J)**. RV = right ventricle, LV = left ventricle, ROCKDNG = *ROCKDN* ^*Gata5-Cre*^ mutants. Scale bar = 100 µm **(A, D)**, 50 µm **(B, C, E, F)**.

To investigate if proliferation could be the underlying cause of the thin noncompacted walls, the pregnant dams were injected with BrdU, which incorporates into the DNA of the proliferating cells, and E15.5 embryos were collected an hour later. Transverse sections of the heart were stained with BrdU antibody and counterstained with DAPI (Figures 3A–F). There were significantly reduced total cell counts (all DAPI-positive cells) per region of interest in the compact wall in the left and right ventricles in the *ROCKDN* ^*Gata5-Cre*^ mutants compared to control littermates (Figure 3G). This is corroborated with the significant reduction in ventricular wall thickness in the *ROCKDN* ^*Gata5-Cre*^ mutants (Figure 2H). The proportion of BrdU-positive nuclei in the same regions of interest was significantly reduced in the compact left and right ventricular walls in the *ROCKDN* ^*Gata5-Cre*^ mutants (Figure 3H). ROCK has been shown to be essential for cell cycle progression (Kumper et al., 2016), so expression of *p21* (*Cdkn1a*), a cyclin-dependent kinase inhibitor which regulates cell cycle progression at G1, was measured by qPCR. *p21* levels were significantly increased in *ROCKDN* ^*Gata5-Cre*^ mutant hearts at E15.5 (Figure 3I). However, by E17.5, the levels of *p21* expression were comparable between the control and *ROCKDN* ^*Gata5-Cre*^ mutant hearts (Figure 3J), suggesting that this was a temporary disruption in cell cycle progression.

### 3.2 Postnatal recovery of the foetal noncompaction ventricular phenotype

**FIGURE 4:**
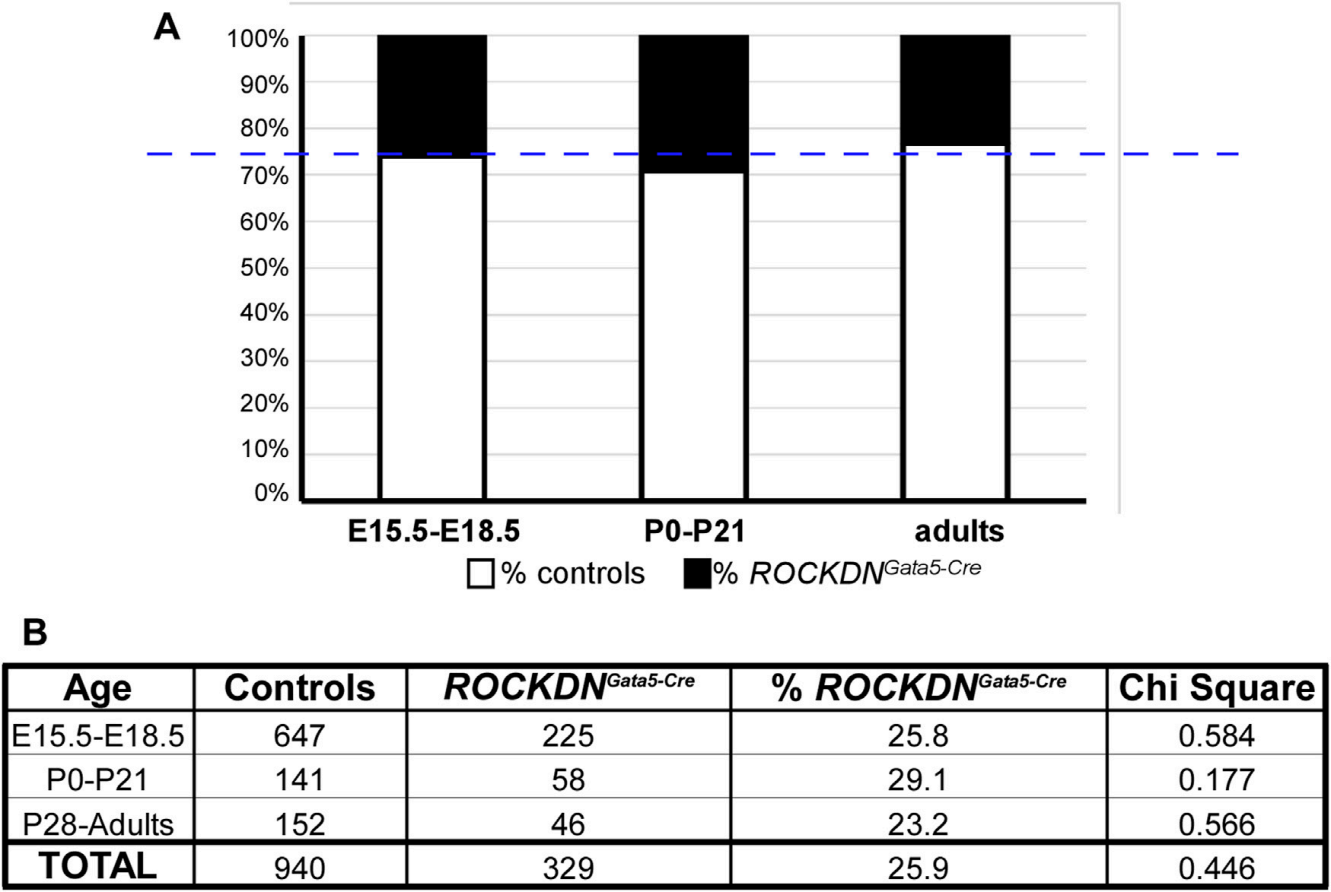
*ROCKDN* ^*Gata5-Cre*^ mutants survived to adulthood in the expected Mendelian ratios. **(A)** In each litter, ∼25% of mice (blue dashed line) were expected to be *ROCKDN* ^*Gata5-Cre*^ mutants, which was very similar to the % of *ROCKDN* ^*Gata5-Cre*^ mutants collected (black portion of the bar graph). **(B)** During development (E15.5–E18.5), postnatally (P0–P21), and in adulthood (P28–15 months), the total number of control and *ROCKDN* ^*Gata5-Cre*^ mutants are shown in the table, and there was no significant difference in chi-square, confirming there was no loss of *ROCKDN* ^*Gata5-Cre*^ mutants.

**FIGURE 5:**
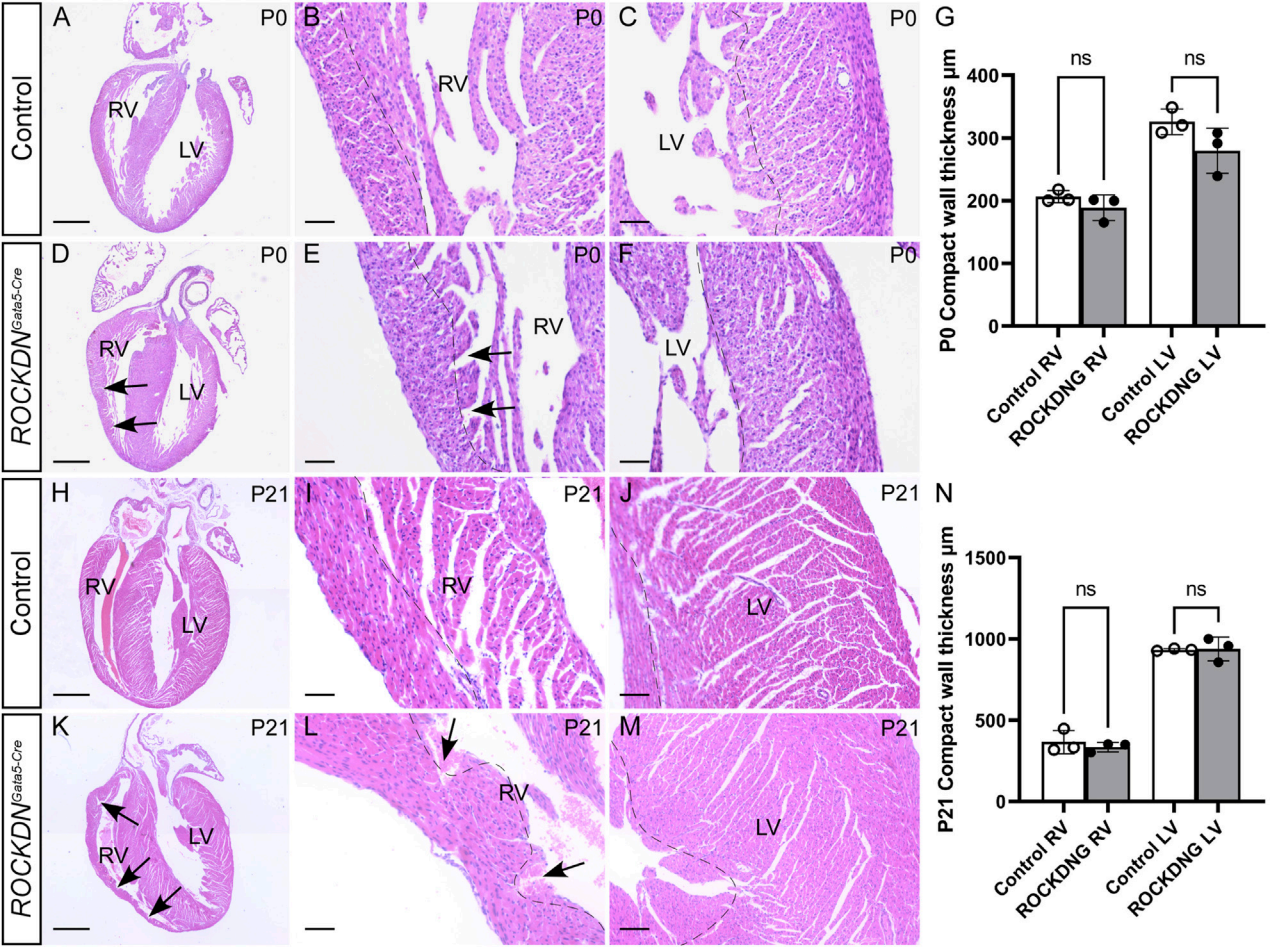
Recovery of ventricular compact myocardial wall thickness after birth. Hearts were dissected from postnatal pups P0 **(A–F)** and P21 **(H–M),** and four-chamber view sections were stained by H&E. The thickness of the compact myocardium (dotted line) in the RV and the LV was comparable as there was no significant difference at P0 **(G)** or P21 **(N)** (*n* = 3). Trabecular myocardial crypts (arrows) were still present in the RV of the *ROCKDN* ^*Gata5-Cre*^ mutants. Data are represented as mean ± SEM. ns (non-significant) by one-way ANOVA with Bonferroni correction for multiple comparisons. RV = right ventricle, LV = left ventricle, ROCKDNG = *ROCKDN* ^*Gata5-Cre*^ mutants. Scale bar = 1,000 µm **(A, D, H, K)**, 100 µm **(B, C, E, F, I, J, L, M)**.

Despite the defects in the ventricular wall observed in *ROCKDN* ^*Gata5-Cre*^ mutants at E15.5, there was no embryonic or postnatal lethality, as the expected number of mutants were identified at the foetal and postnatal stages (overall chi-square *p* = 0.446, Figures 4A, B), suggesting the cardiac phenotypes in the mutant embryos were compatible with survival. In hearts collected on the day of birth, postnatal day (P)0, there was no significant difference in thickness of the compact wall of the right or left ventricle (Figures 5A–G) and this was maintained at P21 (Figures 5H–N), although myocardial crypts were still evident in the RV wall of the *ROCKDN* ^*Gata5-Cre*^ mutants (arrows in Figures 5D, E, K, L).

**FIGURE 6:**
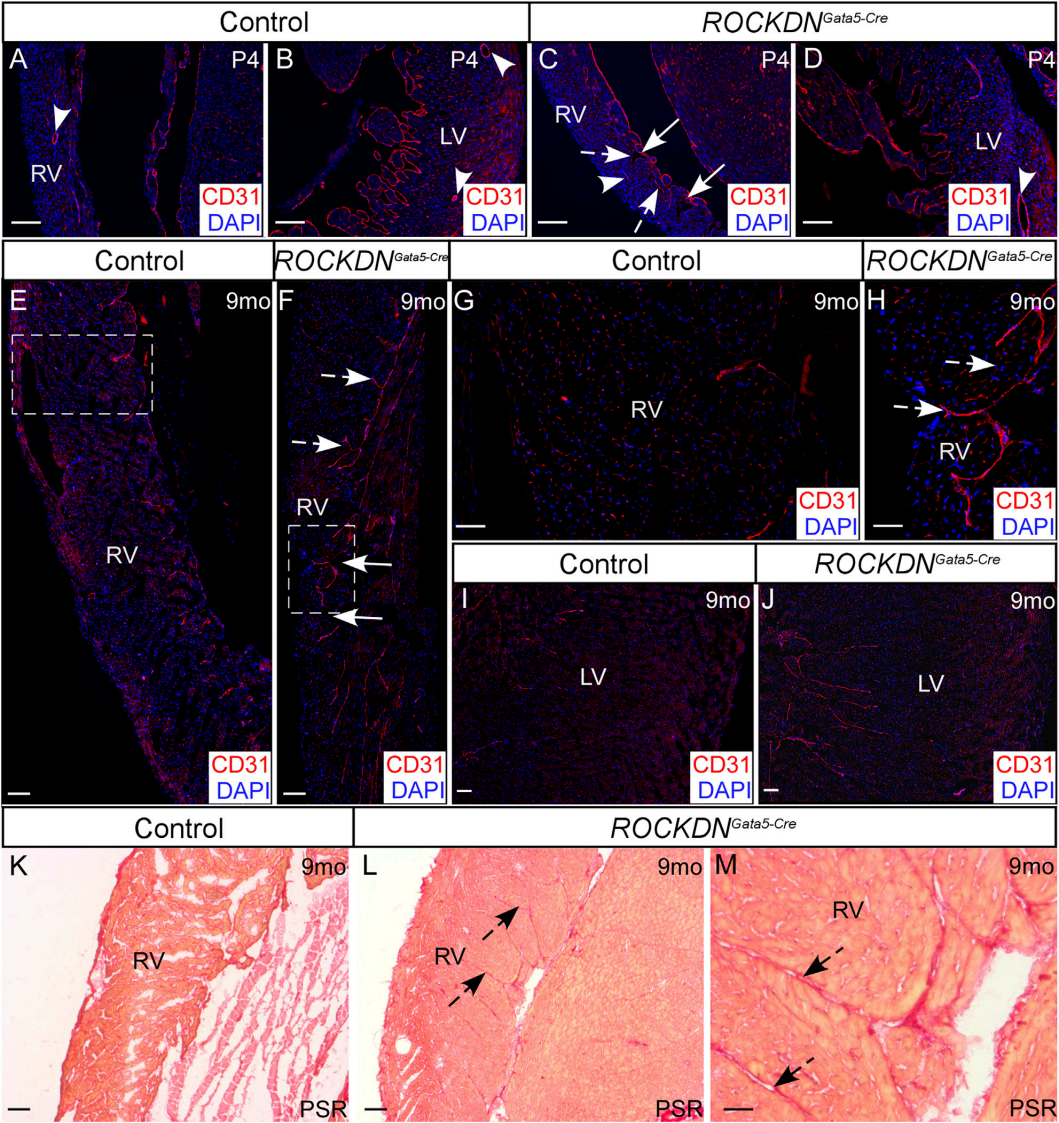
Retention of endocardial cells in myocardial crypts in the right ventricle of the *ROCKDN* ^*Gata5-Cre*^ mutants. **(A–D)** At P4, immunofluorescent staining with CD31 (red), counterstained with DAPI (blue), showed normal maturation of coronary arteries (arrowheads) within the compact myocardium of both ventricles in the controls and *ROCKDN* ^*Gata5-Cre*^ mutants (*n* = 3). Trabecular myocardial crypts were still present in the RV of the *ROCKDN* ^*Gata5-Cre*^ mutants [arrows in **(C)**]. **(E–J)** At 9 months, the control hearts had weak CD31 staining **(E, G, I)**, whereas in the RV of the *ROCKDN* ^*Gata5-Cre*^ mutants (*n* = 5), there was retention of CD31 + ve endocardial cells in the endocardium and between ‘trapped/retained’ trabeculae [dashed arrows **(C, F, H)**]. **(K–M)** Picrosirius Red (PSR) staining showed an increase in fibrosis in the RV of the *ROCKDN* ^*Gata5-Cre*^ mutants corresponding with the retained CD31 staining [black dashed arrows **(L, M)**]. RV = right ventricle, LV = left ventricle, mo = months, PSR = Picrosirius Red. Scale bar = 100 µm **(A–F, I–L)**, 50 µm **(G, H, M)**.

At P4, the morphology of the left ventricular wall was indistinguishable between the control and *ROCKDN* ^*Gata5-Cre*^ mutant hearts, and coronary artery expression of CD31 was visible in both ventricles (arrowheads in Figures 6A–D). The early morphological differences observed in the right ventricle and the myocardial crypts (Figure 2O), however, were still present in the adult *ROCKDN* ^*Gata5-Cre*^ mutants at 9 months (arrows in Figures 6C, F). There was abnormal retention of CD31 staining in the endocardial cells of trabeculae-like structures and on the inner surface of the right and left ventricles (dashed arrows Figures 6C, F, H, J) compared to the control heart (Figures 6A, E, G, I) at P4 and at 9 months. Additionally, in the *ROCKDN* ^*Gata5-Cre*^ mutant hearts at 9 months, an increase in fibrosis in the right ventricle, shown by Picrosirius Red staining, was observed in a similar region to the CD31 staining (dashed arrows in Figures 6K–M). No differences in the level of fibrosis were observed in the left ventricle (data not shown).

**FIGURE 7:**
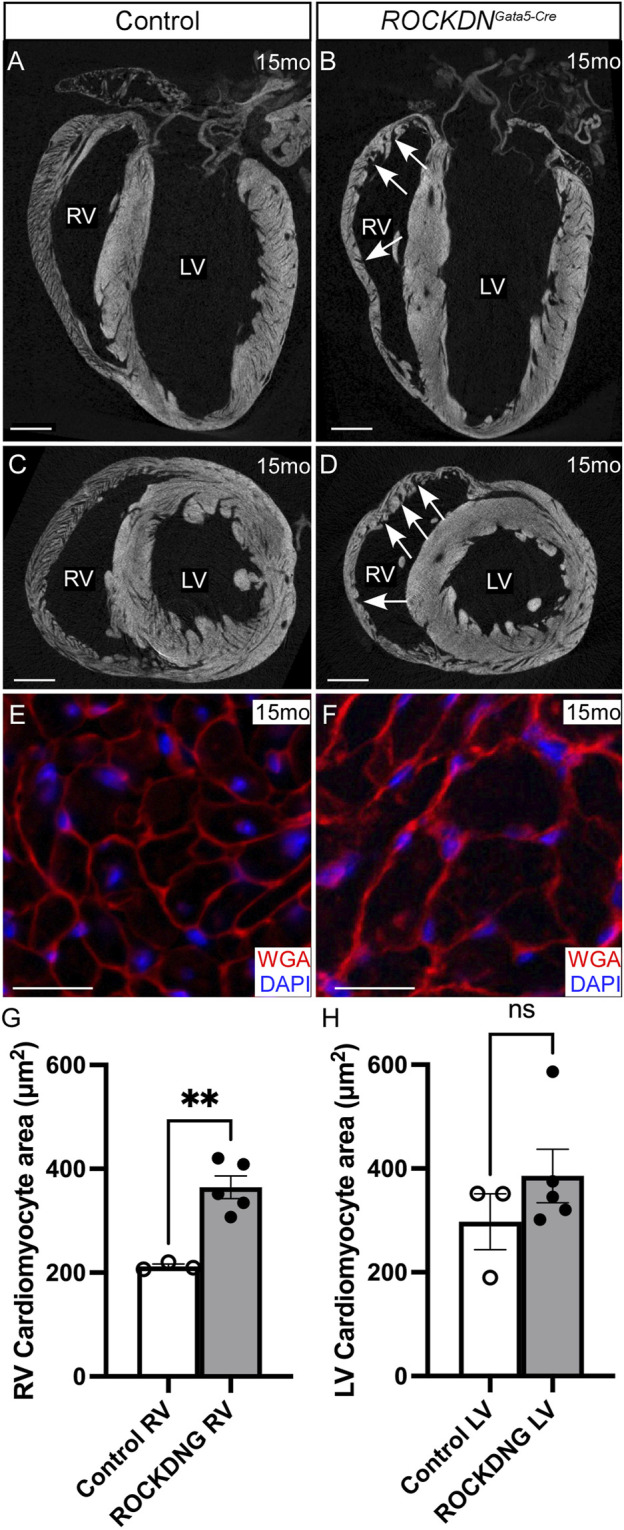
Cardiomyocyte hypertrophy in adult *ROCKDN* ^*Gata5-Cre*^ mutants. **(A–D)** Micro-CT images of 15-month hearts showed that there were no differences in the LV between the control and *ROCKDN* ^*Gata5-Cre*^ mutant hearts, whereas the trabecular myocardial crypts in the RV were retained in the *ROCKDN* ^*Gata5-Cre*^ mutants [arrows in **(B, D)**]. **(E–H)** WGA staining (red), counterstained with DAPI (blue), of 15-month-heart sections visually showed that the cardiomyocytes were visibly larger in the RV of the *ROCKDN* ^*Gata5-Cre*^ mutant hearts **(E, F)**, and this was confirmed by measuring the average area of the cardiomyocyte in each ventricle **(G, H)**, (controls *n* = 3, mutants *n* = 5). Data are represented as mean ± SEM. **p < 0.01 by unpaired t-test. RV = right ventricle, LV = left ventricle, mo = months, ROCKDNG = *ROCKDN* ^*Gata5-Cre*^ mutants. Scale bar = 1 mm **(A–D)**, 20 µm **(E, F)**.

By 15 months, micro-CT images confirmed the left ventricles of the *ROCKDN* ^*Gata5-Cre*^ mutants were comparable with the controls, whereas the right ventricles retained the myocardial crypts but were not visibly thinner (Figures 7A–D). Measurements of the cardiomyocyte area using WGA staining to outline individual cardiomyocytes revealed that in the *ROCKDN* ^*Gata5-Cre*^ mutants, there was cardiomyocyte hypertrophy in the RV wall but not in the LV (Figures 7E–H). Thus, this suggested that the embryonic noncompaction phenotype of the LV was able to self-correct and recover by postnatal stages. However, in contrast, in the RV, there was persistence of the early embryonic phenotype, resulting in abnormal compaction of the trabeculae with the presence of myocardial crypts and increased cardiomyocyte size in the RV of the adult *ROCKDN* ^*Gata5-Cre*^ mutants.

### 3.3 Abnormal morphology of the ventricular walls presented with altered expression patterns of connexins at E18.5

**FIGURE 8:**
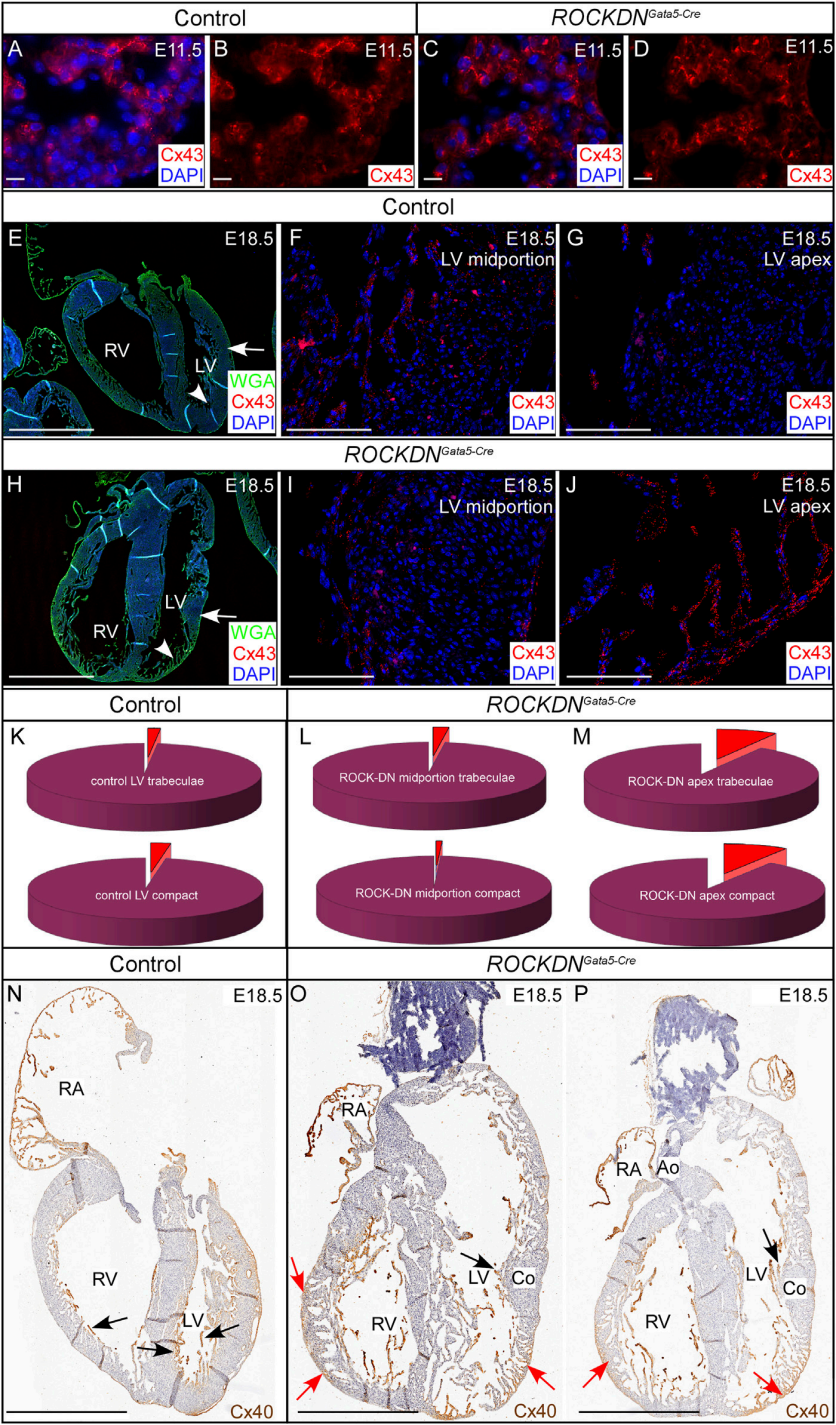
Abnormal Cx43 and Cx40 staining correlates with the partial noncompaction phenotype in the late foetal stages. **(A–D)** E11.5 immunofluorescent staining with Cx43 (red), counterstained with DAPI (blue), was seen throughout the ventricular compact and trabecular myocardium. There were no visual differences between the control and the *ROCKDN* ^*Gata5-Cre*^ mutant hearts. **(E–M)** At E18.5, in the control hearts (*n* = 6) **(E–G)**, sparse Cx43 staining was seen throughout the compact myocardium and trabeculae of the mid-portion [arrow in **(E)**, high power image in **(F)**] and apex [arrowhead in **(E)**, high power image in **(G)**] of the LV wall and the percentage of Cx43 staining (red) in **(F, G)**, was represented by pie charts **(K)**. In the *ROCKDN* ^*Gata5-Cre*^ mutant hearts (*n* = 5) **(H–J)** in the region with a normal compacted wall [arrow in **(H)**, high power image in **(I)**] the percentage of Cx43 staining was very similar to the control staining [compare **(K–L)**]. Whereas, in a region of noncompacted myocardium of the LV apex of the *ROCKDN* ^*Gata5-Cre*^ mutant shown [arrowhead in **(H)**, high power image in **(J)**], there was an abnormal high percentage of Cx43 expression in both the trabeculae and compact myocardium **(M)**. **(N–P)** Cx40 staining at E18.5 shown by Connexin40:eGFP expression (*n* = 3) (visualised by anti-GFP antibody staining, in brown). In the control, Cx40 was strongly expressed in the trabeculae [black arrows **(N)**]. Whereas in the *ROCKDN* ^*Gata5-Cre*^ mutant, Cx40 expression was seen in the noncompacted myocardium of the myocardial crypts of the right ventricle, and there was persisting abundant expression in the thin compact wall and trabeculae [red arrows **(O, P)**]. In the regions of normal compaction (Co), Cx40 had correctly become restricted to only the trabeculae [black arrows in **(O, P)**]. RV = right ventricle, LV = left ventricle, RA = right Atrium, Co = compact myocardium, Ao = aorta. Scale bar = 20 µm **(A–D)**, 1 mm **(E, H, N, O, P)**, 100 µm **(F, G, I, J)**.

Gap junctions are critical for cardiomyocyte cell communication and comprise connexins that are known to play a key role in cardiac physiology. To determine if the abnormal ventricular wall morphology observed in the *ROCKDN* ^*Gata5-Cre*^ mutants affected the expression pattern of the gap junctions, we investigated the expression of the core proteins within these junctions, Cx43 and Cx40. At E11.5, Cx43 was expressed throughout the ventricular compact and trabecular myocardium, and no differences in expression patterns were observed between control and *ROCKDN* ^*Gata5-Cre*^ mutants (Figures 8A–D). By E18.5 (Figures 8E–J), differences in Cx43 expression were evident in the *ROCKDN* ^*Gata5-Cre*^ mutants. In the regions of the ventricular wall where the compact myocardium had remained thin with prominent trabeculae (arrowhead in Figure 8H), there was a higher percentage of Cx43 staining (Figures 8J, M). The mid-portion of the LV, however, showed normal development of the ventricular wall as the compact layer had thickened with no prominent trabeculae (arrows in Figures 8E, H). This region had sparse Cx43 staining, comparable to Cx43 staining throughout the ventricular wall of the control hearts (compare Figures 8F, I, K, L).

To visualise changes in Cx40 expression in the noncompacted myocardium and the ventricular conduction system at later stages, we crossed the *ROCKDN* mice to *Cx40:eGFP* mice (Miquerol et al., 2004). In normal development, initially, Cx40 was widely expressed throughout the ventricular wall, but as the ventricular wall developed, Cx40 became restricted to the inner trabeculae that give rise to the Purkinje network, as seen in the control heart at E18.5 (black arrows in Figure 8N). In contrast, in the *ROCKDN* ^*Gata5-Cre*^ mutant, there was a homogeneous expression of Cx40 across the trabecular layer with atypical retention of Cx40 staining within the thin non-compact myocardium (red arrows in Figures 8O, P). In the thicker, normal compacted regions of the *ROCKDN* ^*Gata5-Cre*^ mutant ventricular wall, Cx40 was correctly restricted to the trabeculae (black arrows in Figures 8O, P). Thus, atypical myocardial Cx43 and Cx40 expression correlates with regions of noncompacted myocardium showing a high intensity of gap junctions.

In postnatal hearts, anti-Cx43 staining showed no visual or quantitative differences at P30 or 15 months (data not shown). Similarly, Cx40:eGFP expression visualised by whole-mount confocal examination of postnatal hearts (*n* = 6 per group) showed normal morphology of the ventricular conduction system in the *ROCKDN* ^*Gata5-Cre*^ mutant hearts (data not shown), correlating with the gradual resolution of the noncompaction phenotype.

### 3.4 Abnormal wall architecture and connexin expression correlated with altered impulse propagation at E18.5

**FIGURE 9:**
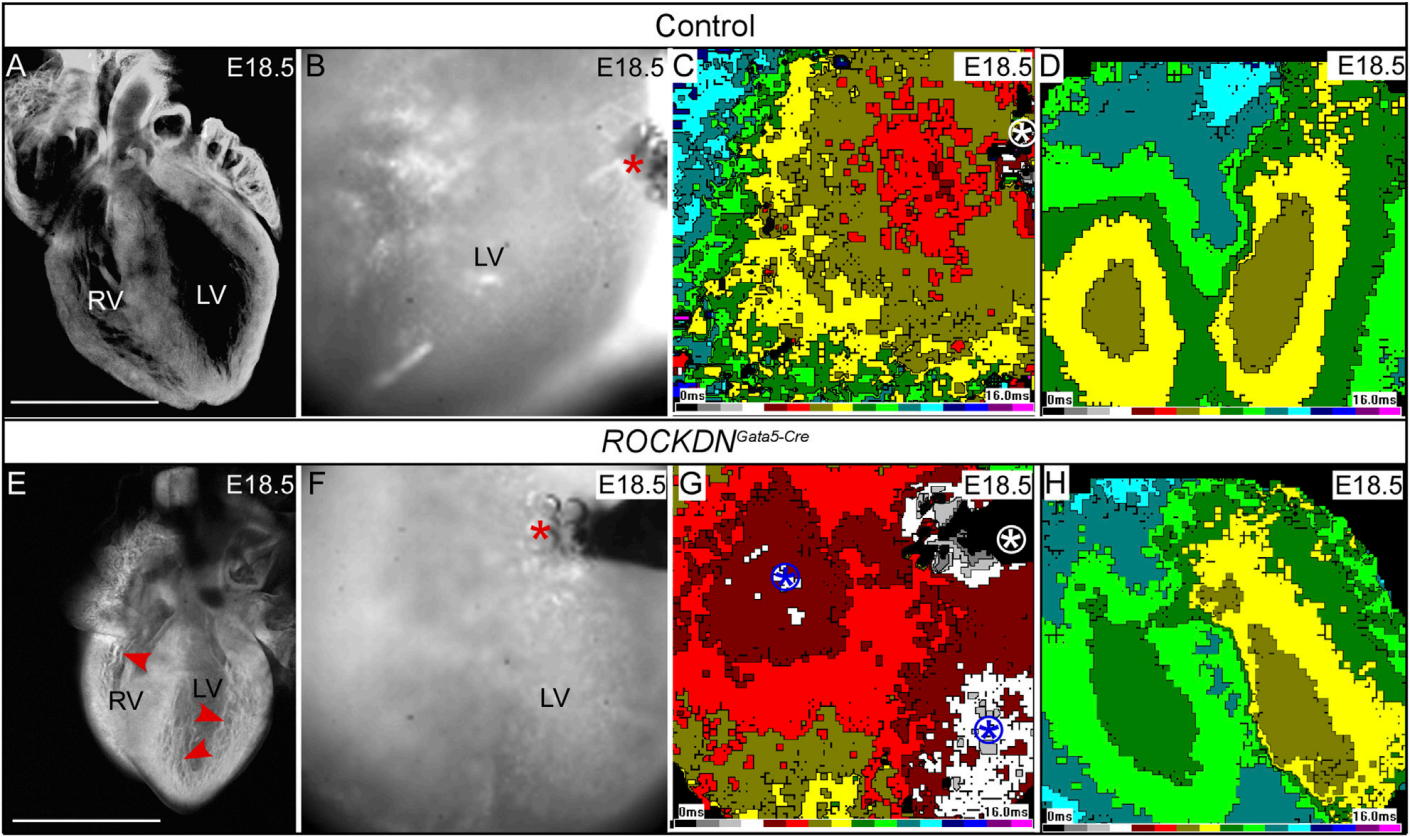
Optical mapping shows abnormal breakthrough during ectopic stimulation of the thin ventricular wall. **(A, E)** E18.5 hearts were visualised by the whole-mount confocal microscopy at ×4 magnification and showed that the control heart **(A)** had a compacted ventricular wall whereas fine abundant trabeculae were still evident in the *ROCKDN* ^*Gata5-Cre*^ mutant [red arrowheads in **(E)**]. The higher-power images from the optical mapping camera show the position of the stimulating electrode, and active pacing was evidenced by the presence of bubbles (red asterisk) **(B, F)**. **(C, G)** Activation waves (1-ms intervals, colour time bar shown) spread fairly isotropically from the stimulation site (white circled asterisk) in the control, starting from the brown colour **(C)**. In the *ROCKDN* ^*Gata5-Cre*^ mutant **(G)**, in addition to the stimulation site (white circled asterisk), there were other breakthrough sites (two blue circled asterisks, starting from grey/white). **(D, H)** There are no differences for spontaneous activation; both hearts show two epicardial breakthrough sites corresponding to terminations of the left and right bundle branches, respectively. Scale bar = 1 mm **(A, B)**. Seven of 20 *ROCKDN* ^*Gata5-Cre*^ mutant hearts showed a pattern similar to **(G)**.

Electrophysiological parameters were evaluated by optical mapping to establish if the early noncompaction phenotype impacted impulse propagation in the heart. No differences in ventricular activation pattern and time in spontaneous or paced rhythm were observed in control (*n* = 34) and *ROCKDN* ^*Gata5-Cre*^ mutant (*n* = 16) embryos at E15.5 (data not shown). In E18.5 foetuses, however, a pace-through phenomenon was observed in 35% of the *ROCKDN* ^*Gata5-Cre*^ mutants (7/20; of which four had a severe defect and three had a milder difference) and this was not observed in any control hearts (*n* = 30 controls, chi-square *p* = 0.002; Figures 9A–C, E–G). The pace-through phenomenon manifested as activation of the ventricular epicardial surface at additional sites (circled blue asterisks in Figure 9G) remote from the pacing electrode (circled white asterisk in Figures 9C, G). These were observed at different locations in addition to the site of spontaneous breakthrough, which was identical in both control and *ROCKDN* ^*Gata5-Cre*^ mutants (Figures 9D, H). Specifically, of the 20 *ROCKDN* ^*Gata5-Cre*^ mutant hearts measured, those with a normal pacing pattern had a left ventricular activation time of 9 ± 4 ms (*n* = 13), whereas those with a pace-through phenotype had a shorter left ventricular activation time (6 ± 1 ms, *n* = 7, *p* = 0.1). There were no differences in heart rate *in vitro* or spontaneous activation patterns (data not shown).

**FIGURE 10:**
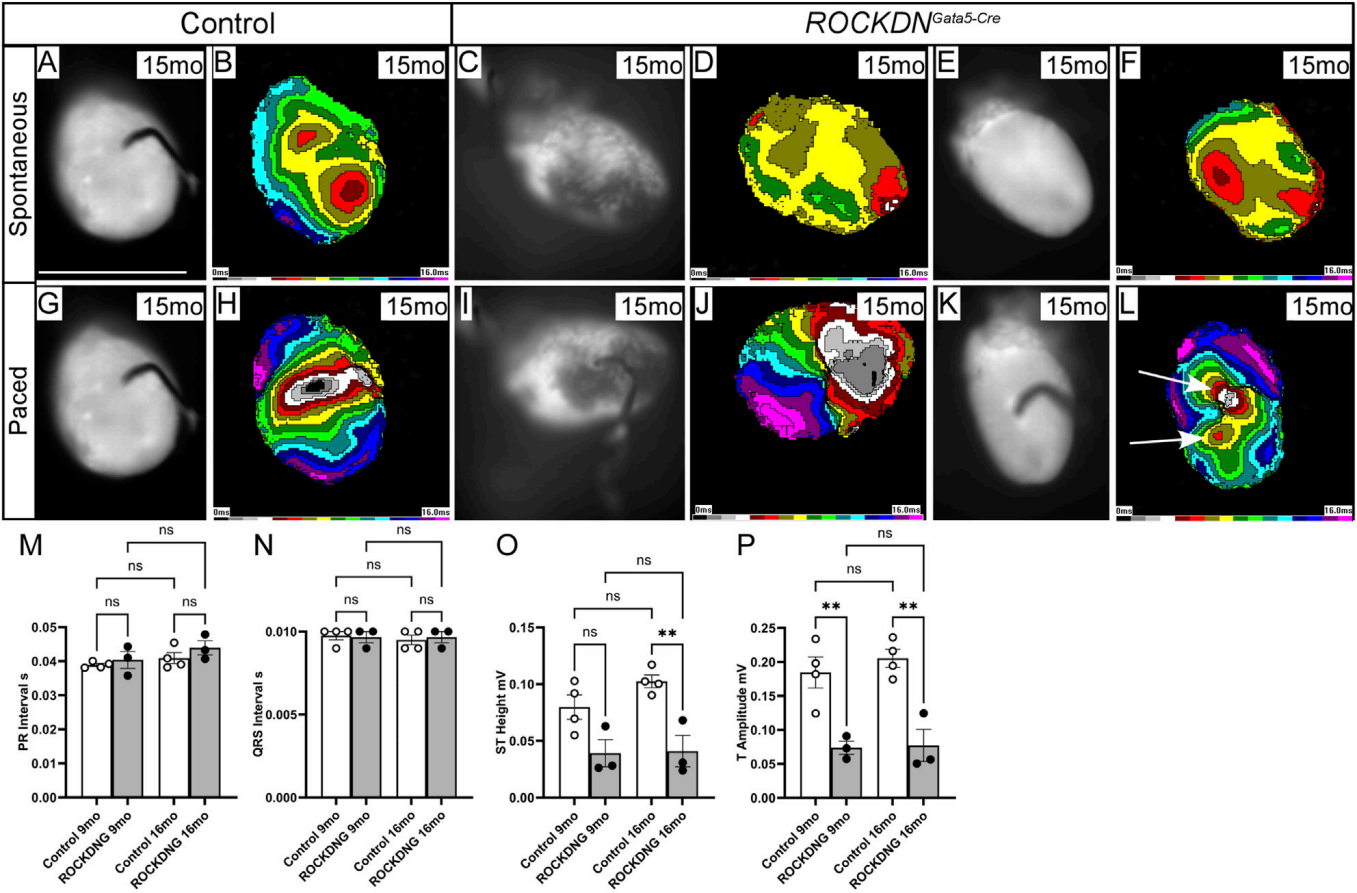
Electrophysiological disturbances in the adult *ROCKDN* ^*Gata5-Cre*^ mutant mice. **(A–K)** Optical mapping showing spontaneous and stimulated activation of the ventricles at 15 months of age. **(A–F)** There was no difference in the spontaneous epicardial activation pattern and the time needed to activate the entire ventricles. **(E–H)** The electrical pacing showed no differences in 5/6 *ROCKDN* ^*Gata5-Cre*^ mutant hearts **(I, J)** compared to controls **(G, H)**. However, in one mutant, a persistence of the ‘pace-through’ phenomenon with additional breakthrough sites was seen [arrows in **(L)**]. The speed of stimulated impulse propagation was not different from controls, as can be seen from comparable isochrone spacing. **(M–P)** ECG traces representing the conduction cycle (averaged over a 60 s period) of control (*n* = 4) and *ROCKDN* ^*Gata5-Cre*^ mutant (*n* = 3) mice at 9 months and 16 months. The PR interval [**(M)**, representing the movement of electrical activation from the atria into the ventricle] and the QRS interval [**(N)**, representing the ventricular depolarisation] were unchanged in the *ROCKDN* ^*Gata5-Cre*^ mutant mice. However, as the heart repolarises, the ST height **(O)** and T amplitude **(P)** were significantly decreased in the *ROCKDN* ^*Gata5-Cre*^ mutant mice at 16 months and at 9 months and 16 months, respectively. Data are represented as mean ± SEM. **p < 0.01 by one-way ANOVA with Bonferroni correction for multiple comparisons. ROCKDNG = *ROCKDN* ^*Gata5-Cre*^ mutants. mo = months, ROCKDNG = *ROCKDN* ^*Gata5-Cre*^ mutants. Scale bar = 1 cm **(A–L)**.

Adult hearts were also analysed by optical mapping at 12 months (*n* = 7 control and *n* = 6 *ROCKDN* ^*Gata5-Cre*^ mutant hearts, data not shown) and 15 months (*n* = 6 control and *n* = 6 *ROCKDN* ^*Gata5-Cre*^ mutant hearts), but no differences in spontaneous heart rate or ventricular activation time with spontaneous pacing were observed between the controls and *ROCKDN* ^*Gata5-Cre*^ mutant hearts (Figures 10A–F). In the paced rhythm, the epicardial activation patterns of the majority of the *ROCKDN* ^*Gata5-Cre*^ mutant hearts (5/6) were the same as in control hearts (Figures 10G–J). However, despite the resolution of the thin left ventricular compact layer phenotype by P30, one mutant displayed abnormal activation in the left ventricular pacing mode (Figures 10K, L).

### 3.5 Abnormal ventricular repolarisation observed in *ROCKDN* ^*Gata5-Cre*^ mutant adult mice

To assess the impact of the early developmental abnormalities in Cx43 and Cx40 expression and myoarchitecture changes on the propagation of electrical signals on the function of the intact adult heart *in vivo*, electrocardiography (ECG) was employed to measure the conduction cycle of the hearts of the 9-month and 16-month controls (*n* = 4) and *ROCKDN* ^*Gata5-Cre*^ (*n* = 3) mutant mice. There were no obvious outward physical differences between these mice. Based on the ECG traces, both the atrioventricular conduction and ventricular activation were unaffected in the *ROCKDN* ^*Gata5-Cre*^ mutant mice (Figures 10M, N), whereas ST height and T amplitude were significantly decreased in the 16-month mutant mice compared to control mice (Figures 10O, P), which may suggest that there was a subtle defect in ventricular repolarisation.

## 4 Discussion

In this study, we investigated noncompaction during embryogenesis and how these early alterations in the myoarchitecture impact the maturation of the ventricular wall and the impulse propagation in the heart. To do this, we utilised the *ROCKDN* transgenic mouse model to target the downregulation of ROCK in cardiomyocytes using the *Gata5-Cre* line and found that at E15.5, mutant embryos had a noncompaction phenotype, and this correlated with a significant increase in *p21* expression and a corresponding decrease in cell proliferation. By E18.5, due to the patchy expression pattern of *Gata5-Cre* within the ventricular wall, regions of the wall remained noncompacted, whereas neighbouring regions developed normally and thickened. This provided a perfect model to compare the impact wall thickness had on conduction. Thin regions of the ventricular wall correlated with the conduction of the ectopically paced beat and increased abnormal expression of Cx40 and Cx43. Interestingly, this model showed the ability of the heart to recover from early structural defects and to different extents in each ventricle.

### 4.1 Changes in ventricular wall architecture

Numerous embryonic mouse ventricular noncompaction models have been described to date, and they are generally lethal around E14.5 [reviewed (Sedmera et al., 2000; Wessels and Sedmera, 2003)]. In contrast, those involving only a portion of the ventricle, such as Notch pathway mutants (Luxan et al., 2013), survive postnatally, more closely mimicking the situation in human patients. Those studies pointed out several factors that are necessary for the proper development of the ventricular compact layer, which is crucial for the pressure-generating function of the left ventricle in particular.

More recent studies in humans suggested that the developmental mechanism leading to a thin ventricular wall phenotype is intrinsic to the compact layer itself and is actually a compact layer proliferation problem (Faber et al., 2021a; Faber et al., 2021b) instead of a failure in the compaction of the ventricular trabeculae. The authors corroborated their point by stereological measurements of absolute volumes of compact and trabeculated myocardium and found no precipitous drop in trabecular volume. Similar trends in absolute compact layer and trabecular volumes were shown also in mice (Jensen et al., 2024). Our study fits nicely with this concept, as we have shown a temporary decrease of proliferation activity, consistent with the known role of ROCK, which is compensated over time, where the proliferation decreases from the peak values of the pre-septation period [reviewed by Sedmera and Thompson (2011)]. A reduction in proliferation at E15.5 correlated with the significant thinner patches of ventricular wall observed in the embryonic hearts in the *ROCKDN* ^*Gata5-Cre*^ mutants, mimicking a noncompaction phenotype. However, this cessation of proliferation was temporary and reversible as cell cycle arrest was no longer apparent by E17.5. No postnatal differences in the overall thickness of the ventricle walls were apparent, suggesting this is a model of a self-correcting cardiomyopathy and a novel role of ROCK in cell cycle regulation during cardiac development. While the changes in proliferative activity may be subtle or short-lived, they could translate, over time, into distinct phenotypes. In our current model, it appeared that part of the compensatory mechanism was the retention of trabeculae and an increase in myocyte diameter, that is, hypertrophy, because we found increased myocyte cross-sectional area in the adults, especially in the right ventricle.

### 4.2 Developmental changes in connexin expression and their functional consequences

Expression of Cx40 (Sankova et al., 2012; Benes et al., 2014) and Cx43 (Franco et al., 1998; Franco and Icardo, 2001; Coppen et al., 2003) shows dynamic expression patterns during embryonic development. Cx40 is expressed earlier and in all the chamber myocardium, including the left ventricle compact layer, gradually becoming restricted to the His-Purkinje system; elsewhere, it is complemented (atria) or replaced (working ventricular myocardium) by Cx43. In this context, the high (but not organised) expression of both main connexins in the trabeculae adjacent to the abnormally thin compact layer at E18.5 (Figure 8) was likely enhancing conduction, which, together with the thinness of the compact zone (shortening the distance), could explain the abnormal propagation of the paced beat, while the spontaneous one is conducted normally.

Similarly, abnormal/delayed differentiation of the thin compact zone was evidenced by persistent Cx40 expression (Figure 8). This is the first study that analysed the myocardial conduction properties of the noncompacted ventricular wall during development. While the defect was eventually recovered morphologically, the conduction phenotype partly persisted despite no detectable changes in connexin protein expression in the ventricular myocardium in adults. However, all the changes found in the adults, including the ECG anomalies, were relatively subtle and did not interfere with animal survival.

### 4.3 Different forms of cardiomyopathy

In familiar cardiomyopathy, it has been observed that the same mutation can sometimes manifest as hypertrophic cardiomyopathy (HCM), dilated cardiomyopathy (DCM), or noncompaction in some individuals (Cahill et al., 2013). The different clinical outcomes may stem from structural differences occurring during development, as highlighted by the *ROCKDN* ^*Gata5-Cre*^ model. During development, the LV had clear regions of normal wall maturation, whereas some regions had very thin noncompacted zones due to the patchy Cre expression of *Gata5-Cre*. As approximately 50% of the LV had normal ROCK expression, it appeared that this allowed the LV to recover and was anatomically normal postnatally. However, the RV had a higher percentage of Cre expression, and a larger proportion of cardiomyocytes had reduced ROCK function. Thus, the RV was not able to recover fully, as it was structurally abnormal because deep myocardial crypts had persisted in the adult *ROCKDN* ^*Gata5-Cre*^ mutant mice. These myocardial crypts are visible from early development, and they normally disappear during the maturation of the ventricular wall. They may result from the incomplete compaction of the trabeculae, as there was evidence that the surrounding endocardial cells had been abnormally retained between neighbouring trabeculae and were consequently incorporated into the compact myocardium. The thickening of the RV wall may have occurred through a compensatory mechanism as the cardiomyocytes were hypertrophic. Interestingly, structural abnormalities such as myocardial crypts and abnormal trabeculae patterns have been suggested to predispose to HCM (Captur et al., 2016) and have been identified in preclinical HCM patients with known mutations (Deva et al., 2013; Captur et al., 2014). The *ROCKDN* ^*Gata5-Cre*^ model represents a mild model as the heart was able to recover to retain a healthy cardiac function, whereas the *ROCKDN* ^*TnT-Cre*^ model, where ROCK function was deleted throughout the ventricular wall resulting in uniform thin compact walls, was embryonic lethal.

This suggests that the ability to recover from an embryonic development defect of each ventricle was independent of each other. At E15.5 in the *ROCKDN* ^*Gata5-Cre*^ mutants, both ventricles were significantly thinner and developmentally delayed. However, after birth, the left ventricle recovered and was able to undergo normal wall morphogenesis (i.e., appropriately thick compact layer and normal trabeculae), whereas the right ventricle remained noncompacted in certain regions, shown by the presence of trapped endocardial cells and increased fibrosis, but the coronary arteries within the compact myocardium had formed normally. The different developmental origins of the left and right ventricles, which are the primary heart field and the secondary field, respectively, may explain the differences observed.

### 4.4 Use of the ROCKDN transgenic mouse model for studying ventricular development and function

This resonates with another study utilising the same *ROCKDN* transgenic mouse line but under the control of two different Cre lines: *aMHC-Cre* or the *SM22a-Cre* (Ellawindy et al., 2015). The latter driver gave rise to a form of arrhythmogenic RV cardiomyopathy, identified by fat accumulation in the RV, which became progressively dilated over time and an increase in sudden death from 4 weeks. The phenotype differed from our model due to the expression of the Cre promoter used. Unlike the *Gata5-Cre*, which targeted cardiomyocytes and the epicardium from E9.25 and continues to be expressed in the heart through development as the ventricular wall matures and compacts, the *SM22a* promoter was expressed in the heart tube from E8.0 with expression decreasing in the heart from E10.5, with no cardiac expression by E13.5. Similar to our model, there was a reduction in proliferation shown by BrdU staining at E12.5, and we have shown this was due to an increase in *p21* expression. However, both the *ROCKDN* ^*Gata5-Cre*^ and *ROCKDN* ^*SM22a*^ mutants highlight that the long-term impact of reducing ROCK function in development affects only the RV and that the LV can self-correct.

In the *ROCKDN* ^*SM22a*^ mutants, conduction disturbances were observed at different phases of the cardiac cycle through the atria shown by prolonged PR intervals and widened QRS complexes (Ellawindy et al., 2015), and the hearts showed dilated ventricles and a thickened LV wall with reduced ejection fraction and fractional shortening. We did not observe any functional consequences by MRI (data not shown); however, we showed ECG abnormalities observed in ventricular repolarisation, which represented the end of the cardiac conduction cycle. Ellawindy et al. (2015) observed a severe build-up of fibrosis throughout the RV in the *ROCKDN* ^*SM22a*^ mutants, whereas in our *ROCKDN* ^*Gata5-Cre*^ mutants, the fibrosis was restricted to regions of “trapped” endocardial cells, indicative of incomplete compaction of the trabeculae during the embryonic stages. This difference could be due to the different patterns of ROCKDN expression dependent upon the Cre driver—early and universal with the *SM22a* line vs. mosaic in our *Gata5-Cre* cross (Figure 1). The mature ventricular wall is formed from three layers—the inner myocardial wall, the middle hybrid myocardial zone, and the outer myocardial wall (Tian et al., 2017). The middle layer is formed from the coalescence of the trabeculae cardiomyocytes (and the surrounding endocardial cells) and the expansion of the proliferating compact cardiomyocytes. This suggests that in the *ROCKDN* ^*Gata5-Cre*^ mutants, there was a temporary cessation of the proliferation of the compact cardiomyocytes into the hybrid component of the myocardial wall layer, giving a foetal noncompaction phenotype. The trapped endocardial cells give rise to the coronary endothelial cells and translocate to an intramyocardial location (Tian et al., 2013). Thus, CD31-positive cells were abnormally retained within the ventricular wall as observed in the *ROCKDN* ^*Gata5-Cre*^ mutants, suggesting abnormal coalescence of trabeculae as a result of reduced ROCK function and possibly accounting for the abnormal pace-through phenomenon and ECG abnormalities in the adult hearts.

In the *ROCKDN* ^*Gata5-Cre*^ mutants, loss of ROCK function in early cardiomyocytes led to abnormal increased expression of Cx43 in the regions of thin myocardium at E18.5. In corneal epithelial cells treated with the ROCK inhibitor Y27632, a similar increase in the number of Cx43 gap junctions was observed (Anderson et al., 2002), suggesting that ROCK genes may be required for the regulated assembly of gap junctions. Altered Cx40 and Cx43 have been correlated with impaired conduction and correlated with cardiac disease (Dupont et al., 2001; Severs et al., 2004; Sedmera et al., 2016). The abnormalities in ventricular activation observed in the adult mice have most likely manifested due to the regions of thin myocardium in the noncompaction phenotype during foetal stages that may have led to the alterations in the RV myocardial architecture in the adult hearts.

We observed a novel consequence of disrupted ROCK signalling in cardiac conduction as abnormal ventricular repolarisation was observed in the adult *ROCKDN* ^*Gata5-Cre*^ mutants. Several previous studies have implicated a role for ROCK in cardiac conduction. Interestingly, when the chemical ROCK inhibitor, Y27632, was added to canine blood-perfused atrioventricular node preparations, this study suggested that one function of ROCK may be to facilitate atrioventricular nodal conduction and to control the ventricular repolarisation process (Sugiyama et al., 2002). In chick embryos, utilising the Y27632 inhibitor caused AV block and established that RhoA-ROCK signalling was essential for the maintenance of the myocardial continuity between the sinus venosus and the atrioventricular node (Kelder et al., 2019).

In conclusion, we present a new non-lethal mouse model with a dominant-negative ROCK isoform expressed in a mosaic pattern through the embryonic myocardium using the *Gata5-Cre* driver. The earliest phenotype involves overall thinning of the compact layer with hypertrabeculation at E15.5 due to reduced myocyte proliferation and *p21* expression. However, by E18.5, the phenotype becomes patchy and is almost completely restored after birth, with only deeper intertrabecular recesses persisting in the right ventricle. There were also electrophysiological anomalies correlating with altered connexin expression. Thus, this is the first description of a model of self-correcting embryonic hypertrabeculation cardiomyopathy. It also provides a new insight into the potential pathogenesis of ventricular noncompaction, pointing to reduced compact layer proliferation rather than deficient trabecular compaction.

## Data Availability

The original contributions presented in the study are included in the article/Supplementary Material, further inquiries can be directed to the corresponding authors.

## Supplementary material

SUPPLEMENTARY FIGURE 1 Downregulation of ROCK caused a thin ventricular wall and embryonic lethality in *ROCKDN* ^*TnT-Cre*^ mutants. *TnT-Cre* mice were crossed with the *R26R* ^*eyfp*^ reporter mouse, and transverse sections from control *TnT-Cre* embryos at E10.5 **(A–G)** and E15.5 **(H, I)** were stained with the GFP antibody (green) to label the Cre-positive cells and MF20 antibody (red) to label the cardiomyocytes. TnT-Cre is uniformly expressed throughout the ventricular wall. **(J–Q)** Transverse sections from control embryos **(J–M)** and *ROCKDN* ^*TnT-Cre*^ mutants **(N–Q)** were stained by H&E, and a representative high-power image of the ventricular wall is shown. At E10.5, there were no distinguishable differences between control **(J)** and *ROCKDN* ^*TnT-Cre*^ mutant **(N)** hearts. By E11.5, the ventricular wall was visibly thinner in the *ROCKDN* ^*TnT-Cre*^ mutant with a reduced number of projecting trabeculae [arrow in **(O)**] compared to controls **(K)**. The entire ventricular wall was significantly thinner in *ROCKDN* ^*TnT-Cre*^ mutant hearts at E15.5 [arrows in **(P, Q)**] than the thick compact wall in the control hearts **(L, M)**. **(R)** The expected percentage of *ROCKDN* ^*TnT-Cre*^ mutants was 25%. The number of mutants was significantly reduced by E16.5–E17.5 with no *ROCKDN* ^*TnT-Cre*^ mutants surviving at birth. RV=Right ventricle, LV=Left ventricle. Scale bar = 100 µm **(A–C, H–Q)**, 20 µm **(D–G)**.
