# Supplementary figures and images for: Morphological, electrophysiological, and molecular alterations in foetal noncompacted cardiomyopathy induced by disruption of ROCK signalling
Source: Front Cell Dev Biol. 2024 Oct 7;12:1471751. doi: 10.3389/fcell.2024.1471751 (PMC11491540; doi:10.3389/fcell.2024.1471751)

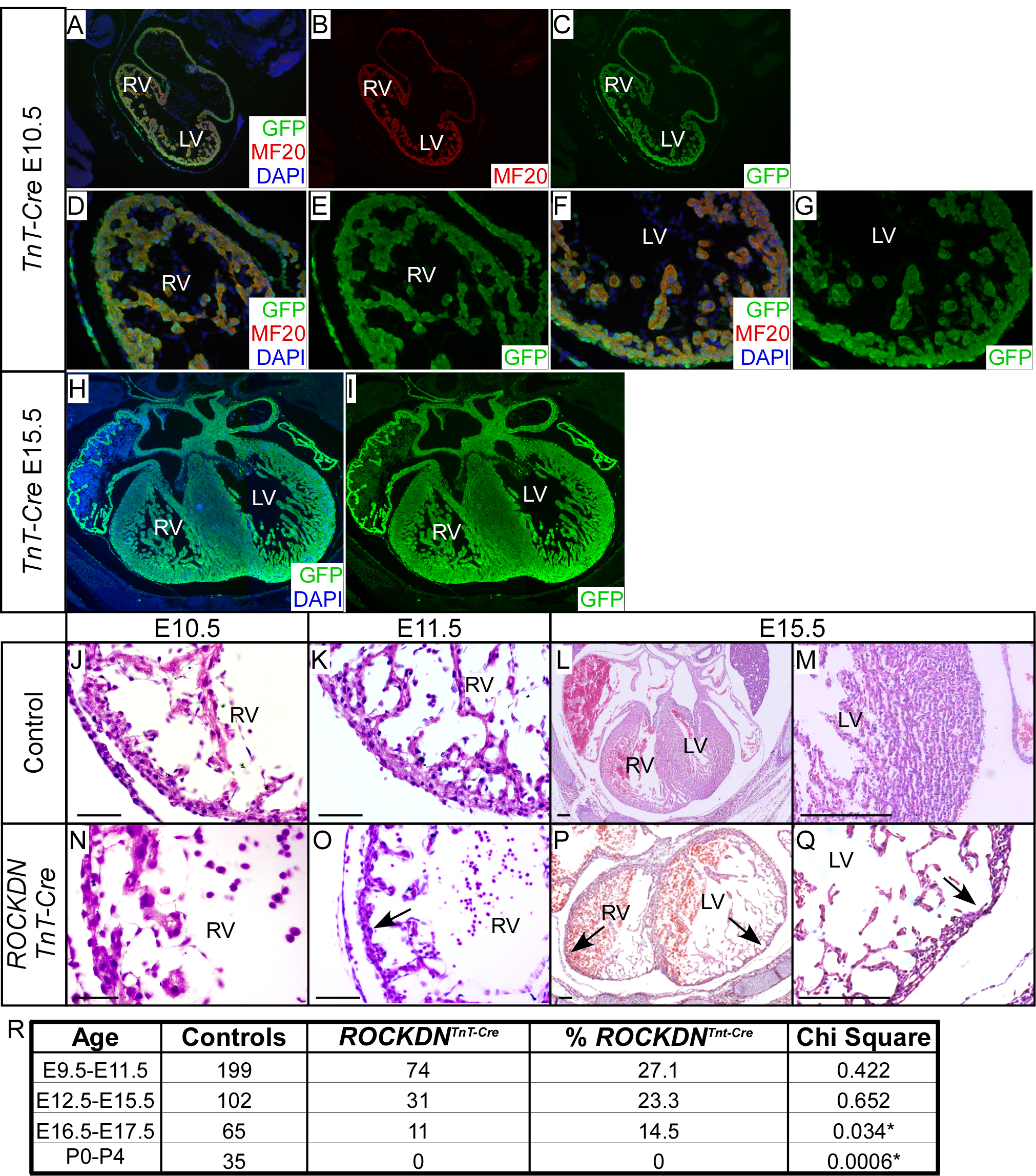

Supplement: Supplementary file 1 [file Image1.TIF]
